# A Scoping Review on Neighborhood Social Processes and Child Maltreatment

**DOI:** 10.3390/bs14121180

**Published:** 2024-12-11

**Authors:** Jisuk Seon

**Affiliations:** Department of Social Welfare, Kyungnam University, Changwon 51767, Republic of Korea; seonjisu@kyungnam.ac.kr; Tel.: +82-55-249-6319

**Keywords:** neighborhood, social processes, child maltreatment, scoping review

## Abstract

Neighborhood contexts, such as structures and social processes, have been explored to understand the etiology of child maltreatment, through the application of an ecological framework in child maltreatment research. While two comprehensive reviews on the relationship between neighborhood structural characteristics and child maltreatment were conducted in the 2000s, no prior study has synthesized the impacts of neighborhood social processes on child maltreatment. This study critically reviews extant literature on the role of neighborhood social processes in child maltreatment by employing the scoping review method, in accordance with the PRISMA-ScR reporting guidelines. The final review included 41 studies between the 1970s and 2022. Findings from the 41 studies were mixed by types of social processes measures, analytic approaches, and types of maltreatment. Future research directions are discussed based on the summary of the key findings.

## 1. Introduction

Child maltreatment is a widespread social problem in the United States, with over 588,000 cases substantiated in 2021, indicating that more than 1600 children are abused or neglected every day [1]. However, these statistics likely underestimate the true scope of the issue, as they only capture instances that have been investigated or substantiated by child protective services (CPS). Over 37% of all U.S. children experience a CPS investigation by the age of 18 [2], highlighting the pervasive nature of the problem. In response, researchers have shifted their focus from examining individual, pathological relationships between parents and children [3] to exploring broader environmental factors that contribute to maltreatment [4], with particular emphasis on neighborhood contexts [5,6,7].

An ecological framework of child maltreatment highlights the roles of environmental characteristics and interactions among children, families, neighborhoods, and larger environments [4,8]. However, it does not explain how or why neighborhoods contribute to relationships between children and parents. Two prominent theoretical frameworks have been used to examine the role of neighborhoods in child maltreatment: social disorganization theory and social capital theory. These illustrate specific pathways by which neighborhoods are more or less likely to result in child maltreatment. Social disorganization theory, originally developed to explain crime and delinquency, suggests that neighborhoods facing structural disadvantages and lacking shared goals, mutual trust, and social control are more likely to experience deviant behaviors [9,10]. According to child maltreatment research based on this theory, neighborhood residents who cannot effectively organize and agree on common values are less likely to intervene when issues like child abuse arise [11]. Social capital theory, on the other hand, emphasizes the importance of social networks and community cohesion in fostering environments that reduce deviant behaviors [6,12,13,14,15]. Strong social capital in a neighborhood enables residents to support one another, create a sense of belonging, and work collectively to address social issues, including maltreatment.

Within the past four decades, studies have examined how both the structural characteristics and social processes of neighborhoods contribute to the likelihood of child maltreatment. Structural characteristics, such as poverty, residential instability, and unemployment, are objective indicators [16] that can directly affect the wellbeing of children [5,6]. Meanwhile, neighborhood social processes refer to the interactions among residents, such as the level of social cohesion, mutual trust, and the ability to collectively address issues [9,10] like maltreatment [5,6]. These social processes can either mitigate or exacerbate the risks associated with negative structural conditions, creating a complex interplay that requires further investigation [7].

While substantial evidence supports the link between neighborhood structural characteristics and increased risk of child maltreatment [17,18,19,20,21,22,23,24,25,26,27,28,29,30], the role of social processes remains an emerging area of research [31,32]. The mechanisms through which social processes impact child maltreatment outcomes are still not fully understood. Neighborhoods with concentrated disadvantages may foster stronger social ties due to the mutual need for support, which could reduce maltreatment by buffering the stress of residents. Alternatively, high stress levels within these neighborhoods could weaken social connections and increase the likelihood of maltreating behaviors. Moreover, social processes themselves might play a direct role in maltreatment or mediate the effects of structural disadvantages. Given these competing possibilities, it is critical to explore the specific ways in which neighborhood social processes influence child maltreatment.

### 1.1. Rationale

Two comprehensive reviews, published more than 15 years ago, advanced our knowledge of the relationship between neighborhoods and child maltreatment [5,6]. However, both reviews focused mostly on neighborhood structural characteristics. This is unsurprising given research on neighborhood social processes was quite thin at the time of publication. Also, both reviews only included studies published by 2004. The authors of each called for more studies on neighborhood social processes that might offer definite mechanisms involved in child maltreatment, such that understandings of how social processes are experienced by families in ways that lead to maltreating behaviors would increase. This is not only theoretically important to explicate pathways to child maltreatment based in neighborhood contexts, but also in practice and policy. For example, understanding these mechanisms might support the implementation of individual- and/or community-level interventions that do not necessarily require the large-scale policy interventions needed to change structural characteristics. Recognizing this, scholars have conducted more research on neighborhood social processes and child maltreatment since 2004. One recent systematic review, published in 2020, critically reviewed the studies between 2008 and 2019 on the impacts of neighborhood collective efficacy on child maltreatment. Findings indicate that while social cohesion as a construct of collective efficacy appeared to be protective for maltreatment, studies on informal social control, the other construct of collective efficacy, were lacking and their findings were inconsistent [11]. However, this study reviewed only certain types of social processes, such as collective efficacy, and selected studies available between 2008 and 2019 only. Therefore, no synthesis of the findings on the relationship between neighborhood social processes and child maltreatment has been conducted.

### 1.2. Objectives

This study critically reviews the extant published between the 1970s and 2022 on neighborhood social processes and child maltreatment to identify possible ways in which individuals interact and patterns of behaviors which can be modified to decrease child maltreatment. Based on this review, I discuss future research directions on the relationships between neighborhood social processes and child maltreatment.

## 2. Methods

### 2.1. Protocol

This study uses a scoping review, a method proposed by Arksey and O’Malley (2005) [33]. While other review methods, such as systematic review, require quantitative quality assessment, a scoping review is typically a narrative presentation that includes both quantitative and qualitative research. It is an appropriate alternative to a systematic review when literature is complex [33,34]. It is appropriate to the current topic because some research on neighborhood social processes and child maltreatment has taken ethnographic and qualitative approaches. A detailed review protocol was developed to outline the objectives, specify the inclusion criteria, and explain the methods for data extraction, based on the stages of the framework for scoping review, suggested by Arksey and O’Malley (2005) [33]. Additionally, this study adopted the framework set forth by the Preferred Reporting Items for Systematic Reviews and Meta-Analyses Extension for Scoping Reviews (PRISMA-ScR) checklist [35] to improve the review’s methodological transparency and rigor.

### 2.2. Search and Information Sources

Articles were identified through a comprehensive literature search using major electronic databases, including Psychinfo, Social Service Abstracts, Sociological Abstracts, PubMed, Google Scholar, and ProQuest. As framed by social disorganization theory and social capital theory, search terms such as child and maltreatment (or abuse or neglect) in combination with neighborhood (or neighbor), community, processes, a combination with social and support, participation, control, cohesion, collective efficacy, and capital were entered to elicit appropriate articles. Specifically, the initial search strategy in the protocol used the query (“Child”) AND (“Maltreatment” OR “Abuse” OR “Neglect”) AND (“Neighborhood” OR “Neighbor” OR “Community” OR “Processes”). The search expanded to include the characteristics of social processes by using the query (“Child”) AND (“Maltreatment” OR “Abuse” OR “Neglect) AND (“Social”) AND (“support” OR “participation” OR “Control” OR “Cohesion” OR “Collective efficacy” OR “Capital”). I used “abuse” and “neglect” as synonyms of “maltreatment” following the definition of child maltreatment in the Federal Child Abuse Prevention and Treatment Act [1]. In addition, I used the broad search term “neighborhood” rather than using specific neighborhood units, including zip code, Census tract, and Census block, so that studies that measured neighborhood social processes by perceptions of individual people without specifically indicating such neighborhood units were all included. Additional relevant articles were obtained by reviewing the references cited in initial search results. The search was performed between August and September in 2023.

### 2.3. Eligibility Criteria and Selection of Sources of Evidence

Peer-reviewed studies as well as masters theses and doctoral dissertations were included if they: (1) were published in English; (2) examined child maltreatment as a dependent variable (including those that aggregated types and those that did not); (3) analyzed at least one predictor for neighborhood social processes; and (4) if quantitative, included bivariate or multivariate analysis. The final search was executed in December 2023.

The initial online search using the search terms yielded 1717 studies. In total, 11 duplicate records of studies were removed, resulting in 1706 studies. The group included many studies that did not actually address neighborhoods but only mentioned them. Therefore, the search was narrowed to studies that included one of these terms for social processes predictors exactly: social support, participation, social control, cohesion, and collective efficacy. This yielded 263. I briefly reviewed these for the inclusion criteria by scanning the abstract, introduction, and tables from each study, which eliminated all but 41. I then reviewed these 41 to eliminate other studies that did not meet the inclusion criteria. Even though I aimed to include studies with any type of child maltreatment as the dependent variable, the search results contained studies with self-reported CPS involvement or official records of CPS involvement as the dependent variable. This might make it challenging to compare findings on the dependent variables, which required additional review. I discussed the points of discrepancies that arose when verifying the inclusion criteria with two colleagues who have expertise on this topic, and ultimately chose to include them in this scoping review, upon consultation. As research on neighborhood social processes and child maltreatment using different sources, such as community surveys, is growing, I concluded that studies examining CPS involvement might have pertinent information on child maltreatment in relation to neighborhood social processes, as suggested in Coulton et al.’s (2007) alternative pathways [5]. Also, some studies suggest that self-reported CPS involvement is a reliable proxy for child maltreatment along with CPS official records [36].

**Figure 1:**
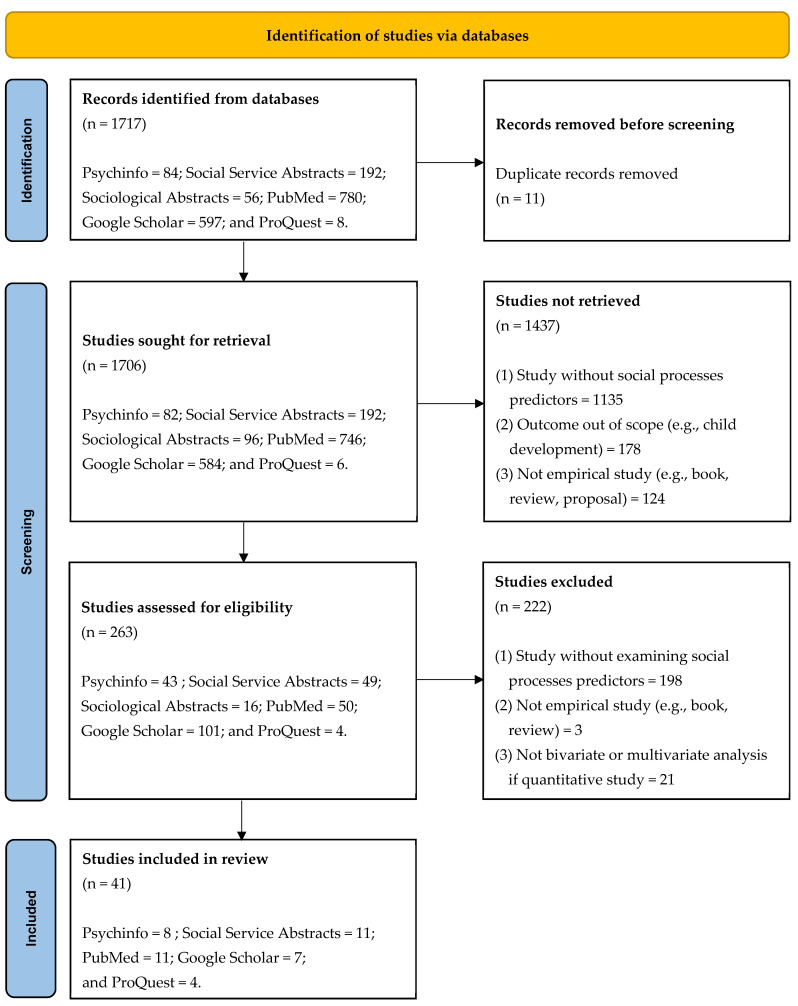
Flow diagram using the PRISMA-ScR guidelines.

Figure 1 illustrates a flow diagram describing the search and selection process, in accordance with the PRISMA-ScR reporting guidelines.

## 3. Results

Table 1, charted by an author independently, lists the selected studies detailing theories that guided the research, sources of data, study samples, analytical approaches, dependent and independent variables including specific measures, and main findings. The last column of the findings indicates which social process characteristics are protective or pose a risk for child maltreatment in each study. These 41 studies provide several insights. First, a growing number of studies have been designed to explain the role of neighborhood social processes in child maltreatment by developing and implementing community survey methods. Second, findings in the observed relationships between neighborhood social processes and child maltreatment are mixed in terms of measures of social processes and type of maltreatment. Last, the application of analytical approaches designed to deal with the statistical issues posed by social processes among individuals that are nested within neighborhoods has advanced. The discussion below amplifies these insights.

### 3.1. Data and Sample

“Data” and “sample” columns in Table 1 provide information about sample type and size and neighborhood unit (if identified) for each study. Out of 41 studies, over 78% (n = 32) used quantitative data. Most of these (n = 20) used original community survey data [24,31,40,43,47,48,49,52,53,57,58,59,61,63,64,70,71,73,74]. The others (n = 12) used secondary data that had already been collected and made publicly available: the National Study of Child Abuse and Neglect in South Korea [37], the Fragile Families and Child Wellbeing Study (FFCWS) [7,32,38,39,56,67,68], the National Longitudinal Study of Adolescent Health (Add Health) [55], and the Project on Human Development in Chicago Neighborhoods (PHDCN) [29,66]. The others (9) used qualitative data, such as observations and interviews [21,22,44,45,46,50,51,54,72]. Over 75% of the studies (n = 31) were conducted in the United States. The rest (n = 10) were conducted elsewhere, including in China [73], Japan [48], South Korea [37,40,44], Vietnam [43], Russia [44], Colombia and Spain [52], Australia [72], and Israel [70]. Six studies focused on fathers and mothers [31,43,44,47,52,74] while the rest focused on mothers only.

### 3.2. Measures of Neighborhood Social Processes

Among the studies which specified theories that guided their research to scrutinize neighborhood social processes and child maltreatment, the majority relied on either an ecological framework or social disorganization theory, or they used both, with some exceptions (e.g., [53]—Environment–place duality framework; [54]—social capital theory; [36]—social learning theory, family coercion theory; [64]—social capital theory, family stress theory; [71]—intersectionality theory; and [67]—risk and resilience perspectives) depending on their focus of research. In line with their use of social disorganization theory, most studies used three types of factors to gauge neighborhood social processes that have been hypothesized to be inversely associated with child maltreatment: collective efficacy, which is represented by informal social control and social cohesion and trust, social network and community participation [10]. Some studies examining collective efficacy examined it jointly with informal social control and social cohesion and trust [29,31,32,39,53,66,68,69], while others investigated these constructs separately [7,36,37,38,40,43,56,57,73]. Eleven studies included other social processes factors that are considered to affect child maltreatment: various types of social support (i.e., family, friend, emotional, tangible, or companionship [37,47,64,74]), social resources [51,55], neighborhood qualities [24,49], demands of social readjustment (i.e., life events that require adjustment such as stress) and use of helpers in response to those demands [51], use of public facilities as an indicator of interaction between residents in neighborhood [61], social capital [48], and perceptions on retaliation and victimization [69]. Community participation is the least measured (n = 4) [39,52,61,68].

Results from the relationships between neighborhood social processes and child maltreatment were inconsistent as they looked at different types of social processes and of maltreatment. Only four of the forty one studies assessed more than two types of social processes factors together in the analyses across various types of child maltreatment and found that social processes lowered child maltreatment risk [48,50,51,64]. Thus, only those four studies serve to validate social processes as protective factors for actual child maltreatment or potential to abuse, despite a variety of ways in which social processes type and maltreatment were measured. Fujiwara et al. (2016) [48] investigated 1277 women with 4-month-old infants in Japan and found that both neighborhood capital (i.e., trust) and social networks were negatively associated with the use of infant physical abuse. In this study, neighborhood capital was measured by one question: “Do you think that people in your neighborhood trust each other?” with a four-point Likert scale response, ranging from yes to no. Social network was measured by two questions with two response options, yes or no: “Do you have someone to consult with in the community?” and “Do you have someone who can help you with child-rearing in the community?”. Merritt (2009) [64] examined 400 parents in Cleveland and found that both family support and friends support were related to lower Child Abuse Potential Inventory (CAP) scores [75]. In this study, family and friends support was measured by the Multidimensional Scale of Perceived Social Support with 12 items. Two earlier qualitative studies comparing neighborhoods with higher risk versus lower risk of child maltreatment found that families living in neighborhoods with lower risk described all aspects of social processes, which were asked in interviews, in positive ways in general, including neighborhood as a resource, a greater number of people in their network, a better place to raise children, etc. [51]. Community leaders and service clients in neighborhoods where child maltreatment rates were rising expressed their neighborhood social processes negatively, however, indicating they considered quality of life in the neighborhood poor and people in the neighborhood not particularly active [50].

However, two studies reported social companionship, a type of social support, as a risk factor, rather than protective, for child physical abuse in relation to parents’ drinking [47] and for child physical abuse by mothers [74]. These contradict the hypothesis of social disorganization theory, meaning that social companionship may put children at risk of maltreatment. Greater interactions and connections between neighbors are generally theorized to enhance the wellbeing of children, but findings from these two studies indicated that parents who had greater companionship were more likely to maltreat their children. Results from qualitative research augmented this finding that, due to social network homophily, disadvantaged parents might be connected to others whose advantages may increase CPS involvement [46]. These imply that not all social processes factors may have protective impacts on child maltreatment.

### 3.3. Measures of Child Maltreatment

In terms of maltreatment type, 23 out of 41 studies examined the effects of neighborhood social processes on physical abuse solely or jointly with other types of maltreatment, followed by neglect, psychological abuse, and sexual abuse. Among these, 17 examined child neglect, of which 8 found no associations with any type of social processes [32,38,39,55,56,59,63,68]. Additionally, only one study investigated sexual abuse in relation to collective efficacy and social network and found negative relationships between them [66].

While the majority of studies (n = 23) measured child maltreatment through self-reports of parental behavior, nine studies analyzed CPS involvement. Out of the 23 studies using self-reports of parental behavior, 18 studies utilized the Conflict Tactics Scale Parent—Child (CTS-PC; [76]) [7,19,31,32,37,38,39,40,47,56,57,58,59,67,68,73,74], including one utilizing it with modifications (Emery et al., 2015) [43]. One study used CAP to score parental behavior [64]. Four studies measured parental behavior of child maltreatment asking parents about how often it happened [48,55,71] or their potential abuse of children using vignettes [44] rather than using any scales. Out of the nine studies measuring CPS involvement, eight used official CPS records [21,22,49,50,51,61,63,66], but one used self-reports of CPS involvement [36]. Finally, one study measured child maltreatment by both CAP score and official CPS records [24].

### 3.4. Analytical Approach

Comparing high-risk versus low-risk areas to understand how neighborhood social processes make variations in child maltreatment by conducting bivariate analyses was more common in the 20th century studies than more recent ones. Four studies employed *t*-test [22], correlation [49] and analysis of variance [52,72]. Three applied qualitative methods, such as description and content analysis [21,50,51].

As secondary data and community surveys have become more available since the 2000s, several different types of statistical regression for estimating the relationships between social processes and child maltreatment have been applied depending on the characteristics of variables. Out of the studies mostly conducted after the 2000s, 17 analyzed social processes at the individual level, focusing on behaviors of individual residents. Three indicated linear regressions [24,61,63], six indicated logistic regressions [36,48,56,70,71,73], and one indicated both logistic and binomial regressions [58]. In order to investigate neighborhood social processes as a whole, five studies included structural equation modeling (SEM) using a latent social processes variable [7,32,38,39,59]. Additionally, one employed a latent growth model based on SEM to estimate growth trajectories of physical aggression in relation to neighborhood cohesion [67].

Out of the studies mostly conducted after the 2000s, 14 analyzed social processes at multiple levels, in line with Coulton et al.’s (2007) and Freisthler et al.’s (2006) recommendations in their two review studies on the effects of neighborhood on child maltreatment [5,6]. This approach handles the clustering issues in statistical analyses, with individuals’ social processes nested in neighborhoods, to better estimate the mechanisms by which neighborhood social processes influence maltreatment of children taking neighborhood structures into account. Specifically, three indicated multilevel linear regressions [29,64,66], four indicated multilevel logistic regressions [37,55,57,74], two indicated multilevel poisson regressions [31,47], three indicated random effects regressions [40,43,44], and one indicated multilevel mixed effects [53]. In addition, one employed SEM by complex model [68].

Three qualitative studies in the late 2010s and 2020s employed thematic content analysis [45] and inductive analysis [46,54] to explore contextual social processes factors of child maltreatment by implementing interviews.

## 4. Discussion

This study highlights a growing body of research on how neighborhood social processes influence child maltreatment, particularly since two earlier reviews [5,6] noted limited literature on the topic. Over 70% of the 41 studies (n = 29) reviewed were conducted after 2010. However, generating conclusive findings remains challenging due to the subjective nature of neighborhood social process measures, which vary across individuals and locations. People’s perceptions of their neighborhood can differ based on experiences and local amenities, which may explain mixed findings on the impact of these processes on child maltreatment. This argument is underpinned by 14 studies that set study locations as narrowly as possible by looking at particular regions in California, Nebraska, Ohio, South Carolina, and Washington State using community surveys [21,24,31,47,49,53,54,57,58,59,63,70,71,74]. Despite differences in findings based on social process types and study locations, further research is needed to explore how neighborhood interactions influence child maltreatment, particularly in suburban and rural areas, which have been underrepresented in the literature [77].

Many studies used the same datasets, but the findings were inconsistent. For example, eight studies using the Fragile Families and Child Wellbeing Study (FFCW) reported varying relationships between social processes and child maltreatment, depending on factors like data waves, sample selection, and analytic approaches [7,32,36,38,39,56,67,68]. Similarly, two studies using the Project on Human Development in Chicago Neighborhoods (PHDCN) reported conflicting results regarding the association between collective efficacy, social networks, and child maltreatment [29,66]. Therefore, more replications of studies examining social processes and child maltreatment in different neighborhoods with similar conditions are also necessary to improve the generalizability of study findings.

Inconsistent measures of neighborhood social processes also contribute to the mixed findings. For instance, informal social control, a key component of collective efficacy, is often measured by respondents’ willingness to intervene in street crime rather than child maltreatment specifically [7,29,31,32,36,37,38,39,53,56,57,58,59,63,66,68,69,73,74]. To address this, recent studies have developed measures of informal social control directly related to child abuse [40,44]. Reflecting the different dimensions of informal social control that are specific to child maltreatment [11], one recent study found that study participants’ responses to suspected child maltreatment are varied; more than 70% would contact authorities only, followed by contact authorities and go to persons involved, other actions, and do nothing [69]. This type of particular information on the relationships between informal social control and child maltreatment may enable future studies to elucidate how to measure social processes measures and how to examine them with child maltreatment.

Similarly, research on social support, which can play a protective role in child maltreatment, has used general measures of support rather than neighborhood-specific support. In total, 11 investigated social support utilizing several different scales, including the social support scale from the Panel Study on Korean Children, a modified Social Provision Scale, the Interpersonal Support Evaluation List, and the Multidimensional Scale of Perceived Social Support [22,24,37,47,48,49,51,59,64,71,74]. One exception is Maguire-Jack and Wang’s (2016) [59] study that used a subset of a scale from the Family Support Study, which was intended to assess family support from friends or neighbors for child neglect [78,79]. More scales to gauge neighborhood social processes that are specifically tailored to measure neighbors and child maltreatment are needed.

While participation in local institutions is thought to promote social cohesion and prevent deviant behaviors, only five studies examined its role in child maltreatment [39,52,56,61,68]. More research is needed to assess whether participation in local institutions can protect against maltreatment.

Qualitative studies provide deeper insights into how various aspects of neighborhood social processes, e.g., environmental quality, neighbor networks, community authority, are associated with child maltreatment in at-risk families [21,45,46,50,51,54]. These studies reveal nuances that quantitative research might overlook, such as how socioeconomic disadvantage influences child protective services involvement.

In considering child maltreatment outcomes, it is important to distinguish between behaviors (e.g., parents’ maltreatment of children) and reports (e.g., neighbor reports to authorities). Coulton et al. (2007) suggested that these two outcomes may involve different mechanisms [5]. For example, the lack of neighborhood control may not affect parents’ behaviors directly but could increase the likelihood of neighbors reporting maltreatment. To date, only one study considered such differences by assessing child maltreatment with two different measures—CAP score and official records of the CPS. While lack of neighborhood control of children was insignificant for the CAP score, it was positively related with reports of child maltreatment [24]. This suggests that the effects of neighborhood social processes may differ by self-reported parents’ maltreating behavior toward their children and maltreatment being reported to authorities by neighbors. Thus, more research is needed to examine how neighborhood social processes influence both maltreating behaviors and reports of maltreatment.

Additionally, understanding how social processes affect specific types of child maltreatment, such as neglect and sexual abuse, is crucial. Despite the prevalence of neglect [1] and sexual abuse [80], studies examining their relationship with neighborhood social processes are limited, and findings often show no significant associations [32,38,39,55,56,59,63,66,68]. The majority of these studies relied on data drawn from the FFCSW that includes very minimal levels of neglect, which is inconsistent with national statistics. In other cases, this may be due to underreporting or the reluctance of neglectful parents to engage with neighbors.

Behavioral pathways, such as parental stress and personal control, may mediate the relationship between social processes and child maltreatment [5]. However, few studies have examined these mechanisms [32,36,38]. Future research should investigate how neighborhood social processes impact child maltreatment through factors such as parental stress and coping.

Finally, the role of time in shaping the relationship between neighborhood social processes and child maltreatment has been largely overlooked [6,72,81]. Longitudinal studies are needed to explore how changes in neighborhood dynamics over time affect the occurrence of maltreatment. While some studies have included length of residence as a control variable [24,31,40,43,56,68], its role in shaping neighborhood social processes remains unclear. In an exception, one study examined the length of residence in the neighborhood on the relationships between collective efficacy and neighbors’ taking actions, but found that it was insignificant [69].

### Limitations

There are several barriers to generating conclusive findings on the impacts of neighborhood social processes and child maltreatment, including variations in subjective perceptions of social processes and neighborhoods, measures for social processes and types of child maltreatment, and analytic approaches. In addition, this review does not assess the qualities of evidence in the studies in any formal sense because it employed the scoping review method.

## 5. Conclusions

This review found that there is progress in understanding the role of social processes in child maltreatment in that more and more studies are examining the question and applying theoretical frameworks that include neighborhood contexts, such as structures and social processes. Despite the varying results across the studies, findings considering the social aspects of individuals’ processes within larger neighborhoods are meaningful, as they can help practitioners design community-based interventions for child maltreatment, in line with Belsky’s (1980) ecological proposal, as a means to prevent and reduce child maltreatment [4]. It is hoped that by comprehensively reviewing the current state of knowledge on the role of neighborhood social processes in child maltreatment, this study will inspire many more studies on this important question. In order to further enhance our understanding, future research should examine how neighborhood structural characteristics affect how families interact in their neighborhoods over time and how this results in child maltreatment, as well as more replications of studies examining social processes and child maltreatment in different neighborhoods with similar conditions.

## Figures and Tables

**Table 1:** Summary of studies on neighborhood social processes and child maltreatment.

Study	Theory	Data	Sample	Analytical Approach ^a^	Dependent Variables	Independent Variables	Findings ^b,c^
1. Ahn & Yoo (2019) [37]	Ecological perspective	2011 National Study of Child Abuse and Neglect in South Korea	N = A total of 4730 Families with children above age three n = 1768 for the younger age group (age 3 to third graders in elementary school) and n = 1774 for the older age group (fourth graders in elementary school and above)	Hierarchical logistic regression	Occurrence of child maltreatment (CM) including physical assault and psychological aggression (scale: Conflict Tactics Scale Parent–Child [CTS-PC])	Ontogenic development: caregiver’s demographic characteristics—age, gender, and education level, psychological traits-depression, stress, and experiences of maltreatment in childhood Microsystem: child’s characteristics—age, gender, preterm birth, and problem behavior by the child. Interactions with their family members—marital satisfaction, experience of partner violence. Perceptions of their own parenting competence Exo-system: Family poverty, community size, informal social control, community cohesion and trust (scale: developed by Sampson et al., 1997 [10]), social support (scale: social support scale from the Panel Study on Korean Children)	1.1. Protective factors: informal social control for both younger age and older age sample 1.2. Risk factors: none 1.3. Insignificant factors 1.3.1. Yonger age sample: community cohesion and trust 1.3.2. Older age sample: social support, community cohesion and trust
2. Barnhart & Maguire-Jack (2016) [38]		2001–2003 Fragile Families Child Wellbeing Study (FFCWS) Wave 3 (child age 3)	N = 1158 Unmarried, non-cohabitating mothers	Structural equation modeling	Physical abuse Neglect (scale: CTS-PC)	Social cohesion Informal social control (scale: developed by Sampson et al., 1997 [10], in the Project on Human Development in Chicago Neighborhoods [PHDCN] study) Mediators: parenting stress, maternal depression	2.1. Protective factors: 2.1.1. Physical abuse: social cohesion, social cohesion → maternal depression 2.1.2. Neglect: social cohesion → maternal depression 2.2. Risk factors: none 2.3. Insignificant factors 2.3.1. Physical abuse: informal social control, social cohesion → parenting stress, informal social control → maternal depression 2.3.2. Neglect: social cohesion, informal social control, social cohesion → parenting stress, informal social control → parenting stress, informal social control → maternal depression
3. Cao & Maguire-Jack (2016) [39]	Social disorganization theory	2001–2003 FFCWS Wave 3 (child age 3)	N = 3288 Mothers	Structural equation modeling	Physical aggression Psychological aggression Neglect (scale: CTS-PC)	Neighborhood (NBH) social processes: Social disorder, informal social control, social cohesion (scale: developed by Sampson et al., 1997 [10]) Internal control Community participation	3.1. Protective factors 3.1.1. Physical aggression: NBH social processes, NBH social processes → internal control, community participation → internal control 3.1.2. Phsychological aggression: NBH social processes, NBH social processes → internal control, community participation → internal control 3.1.3. Neglect: NBH social processes → internal control, community participation → internal control 3.2. Risk factors: none 3.3. Insignificant factors 3.3.1. Physical aggression: community participation 3.3.2. Phsychological aggression: community participation 3.3.3. Neglect: NBH social processes, community participation
4. Coulton et al. (1999) [24]	Ecological framework Social disorganization theory	Community survey 1991, 1992, 1993 CPS data 1990 Census	N = 400 Parents of children N = 20 Census blocks	Hierarchical linear modeling	Child abuse (Scale: Child abuse potential inventory [CAP]—the abuse scale and the experimental neglect scale) NBH rates of CM including substantiated and indicated reports	Individual factors: child abuse in the family of origin (scale: CTS-PC), personal social support (scale: Multidimensional Scale of Perceived Social Support), demographic characteristics, income, education, marital status, tenure in the neighborhood NBH factors: NBH structural factors- Impoverishment, childcare burden, instability, NBH social processes factors (resources and supports)—NBH quality, facilities, disorder, lack control over children	4.1. Protective factors 4.1.1. Total abuse score: individual social support 4.1.2. Experimental neglect: individual social support 4.1.3. NBH rates of CM: NBH facilities 4.2. Risk factors 4.2.1. Total abuse score: none 4.2.2. Experimental neglect: none 4.2.3. NBH rates of CM: NBH disorder, NBH lack control of children 4.3. Insignificant factors 4.3.1. Total abuse score: NBH quality, NBH facilities, NBH disorder, NBH lack control of children 4.3.2. Experimental neglect: NBH quality, NBH facilities, NBH disorder, NBH lack control of children 4.3.3. NBH rates of CM: none
5. Deccio et al. (1994) [22]	Ecological framework	Parent interview (N = 56) in two selected neighborhoods —one with a high rate and one with a low rate—of CPS reports 1988 CPS data 1980 Census	N = 43 Census tracts N = 56 parents in 2 census tracts including one with high CM risk, the other with low CM risk	*t*-test	CM indicated reports including physical abuse, physical neglect, sexual abuse, emotional abuse, and neglect	1. NBH structural factors: total number of persons, income, race, mean age, occupation, education, ratio of family and children in tract, caregiver’s work role, housing characteristics 2. NBH processes factors: perceived personal social support, perceived parenting support (scale: a modified Social Provision Scale)	5.1. NBH with lower CM risk: mean score of personal social support 65.6, mean score of NBH parenting support 26.0 5.2. NBH with higher CM risk: mean score of personal social support 64.8, mean score of NBH parenting support 25.7 5.3. Perceived personal social and parenting support were not significantly different between parents living in high and low risk NBHs.
6. Emery et al. (2014) [40]	Social disorganization theory	2012 Seoul Families and Neighborhoods Study	N = 541 families	Random-effects regression	Severe physical abuse (scale: CTS-PC) Child physical injury	Informal social control of CM (scale: two items from Emery et al., 2013 [41]) Perceived collective efficacy: NBH solidarity, NBH informal social control (scales: developed by Sampson et al., 1997 [10]. and Zhang et al., 2007 [42]) Father’s intimate partner violence (IPV) perpetration (scale: CTS2)	6.1. Protective factors 6.1.1. Severe physical abuse: none 6.1.2. Child physical injury: informal social control × severe physical abuse 6.2. Risk factors 6.2.1. Severe physical abuse: none 6.2.2. Child physical injury: none 6.3. Insgnificant factors 6.3.1. Severe physical abuse: informal social control, NBH solidarity, NBH social control 6.3.2. Child physical injury: informal social control, NBH solidarity, NBH social control
7. Emery et al. (2015) [43]	Social disorganization theory	2012 Hanoi Families and Neighborhoods Study	N = 293 families	Random effects regression	Severe physical abuse (scale: a modified CTS-PC)	Informal social control of CM Perceived collective efficacy: NBH solidarity, NBH informal social control (scales: developed by Sampson et al., 1997 [10], and Zhang et al., 2007 [42]) Father’s IPV perpetration (scale: CTS2)	7.1. Protective factors: protective informal social control 7.2. Risk factors: none 7.3. Insignificant factors: punitive informal social control, NBH solidarity, NBH informal social control
8. Emery et al. (2019) [44]	Social disorganization theory	Interviews in Seoul and Novosibirsk	N = 202 parents n = 100 married men in Seoul n = 102 mothers and fathers in Novosibirsk	Random effects regression	Parent’s potential abuse of children (vignette used)	Having perpetrated physical abuse (scale: a modified CTS-PC) High/low informal social control by neighbors	8.1. Protective factors 8.1.1. Seoul: high informal social control × perpetrator 8.1.2. Novosibirsk: none 8.2. Risk factors 8.2.1. Seoul: none 8.2.2. Novosibirsk: none 8.3. Insignificant factors 8.3.1. Seoul: high perceived informal social control 8.3.2. Novosibirsk: high perceived informal social control, high informal control × perpetrator
9. Finno-Velasquez et al. (2019) [45]	Social disorganization theory	Semi-structured qualitative interview	N = 28 community informants from 18 Census tracts including areas with unusually high or low CM rates	Thematic content analysis	Interview questions As someone who knows this area well, are there things about it that you believe make it particularly [protective against or risky for] child abuse and neglect? Are there things about this area that you believe or that would substantially [decrease or increase] abuse and neglect reporting? Is there anything about the way immigrants relate to each other or other groups/agencies in this area that might contribute to the [higher/lower] than expected occurrence of maltreatment or maltreatment reporting?		CM reporting and behaviors may be related to three themes concerning the socio-cultural context of immigrant communities: cultural norms and values, fear and mistrust of exposure to authorities, and lack of knowledge and misinformation.
10. Fong (2017) [46]		In-depth interviews	N = 40 poor, child welfare-investigated parents	Inductive analysis	Interview questions Tell me her life story in detail, including childhood experiences, housing, employment, experiences with welfare and other social services, and financial strategies. Tell me your own experiences with child welfare, the experiences of others they knew, general perceptions of the child welfare agency and its decision-making, experiences calling the child welfare hotline, and any worries or concerns about child welfare involvement.		Contextual factors of poverty cited in accounts of child welfare involvement: disadvantaged networks, fractural relationships, social service reliance
11. Freisthler et al. (2014) [47]	Ecological-transactional framework of child maltreatment	Community survey 2009 California Department of Alcoholic Beverage Control	N = 3023 parents in 50 cities	Multilevel Poisson model	Physical abuse (scale: CTS-PC)	Alcohol drinking Alcohol outlet density Social support including emotional, tangible support, belongingness (or social companionship support) (scale: interpersonal Support Evaluation List) Attributes of social networks Psychosocial risk factors: depressive symptoms, anxiety, parenting stress	11.1. Protective factors 11.1.1. Alcohol use: tangible support, emotional support 11.1.2. Dose-response: tangible support, emotional support, companionship × off- premise 11.2. Risk factors 11.2.1. Alcohol use: social companionship, companionship × on-premise 11.2.2. Dose-response: social companionship, companionship × on-premise 11.3. Insignificant factors 11.3.1. Alcohol use: average network size, % of local tangible support, % of local emotional support, % of local social companionship 11.3.2. Dose-response: average network size, % of local tangible support, % of local emotional support, % of local social companionship
12. Freisthler & Maguire-Jack (2015) [31]		Community survey 2009 GeoLytics 2009 California Department of Alcohol Beverage Control	N = 3023 parents in 194 zip codes	Multilevel Poisson model	Physical abuse (scale: CTS-PC)	NBH structural factors: families living in poverty, unemployed, female-headed households with children, ratio of children to adults, foreign born who were naturalized, foreign born who are not citizens, Asian/Pacific Islander, longtime residents, recent movers, owner-occupied housing units, ratio of males to females, Hispanic, Black Alcohol outlet density: off-premise outlets, on-premise outlets NBH social processes factors: social disorder, collective efficacy including informal social control and reciprocated exchange (scale: A modified version developed by Sampson et al., 1997 [10])	12.1. Protective factors: collective efficacy 12.2. Risk factors: social disorder, social disorder × number of years living in the neighborhood 12.3. Insignificant factors: none
13. Fujiwara et al. (2016) [48]		Community survey	N = 1277 mothers	Logistic regression	Infant physical abuse	Social capital including perceived neighborhood trust and social support Social network	13.1. Protective factors: social capital, social network 13.2. Risk factors: none 13.3. Insignificant factors: none
14. Garbarino & Crouter (1978) [49]	Ecological perspective	Community survey 1976 CPS data Census	N = 20 County subareas and 93 census tracts	Correlation	Rates of CM reports including child abuse and neglect	Socioeconomic factor: family income Demographic factors: families headed by females, working mothers, families living in current residence less than 1 year Attitudinal factors: perceived importance of support systems and overall perceived quality of NBH life—feeling good about neighborhood, if daycare is important and necessary, if neighborhood is desirable/not desirable Housing: stability score, single family housing, vacant housing Source or CM report: close, distant	In both subareas and census tracts, correlations exised between a factor combined with socioeconomic factor and perceived NBH evaluation.
15. Garbarino & Kostelny (1992) [50]		Interview 1980–1986 CPS data	n = 7 Community leaders and social service clients n = 113 Census tracts n = 4 Communities (2 predominantly African American, 2 predominantly Hispanic)	Description	CM substantiated reports (Types not specified)	NBH structural factors: poverty, unemployed, family characteristics, housing characteristics, race/ethnicity, educational attainment, residential stability Community leader interview questions: How do people see the area? What is the physical appearance of the area? Would you say that the area is stable or changing? How would you describe the quality of life in the area? How active are the people in the area in community activities? List the other agencies that work in the area. What are the major problems in the community?	In area where CM rates were increasing over time, interviewees knew less about community services, demonstrated little evidence of formal or informal networks and supports, reported less positive feeling about political leaders, and had less of a sense of belonging or community.
16. Garbarino & Sherman (1980) [51]	Ecological framework	Interviews with expert informants ranging from elementary school principals to mailmen 1976 CPS data	N = 46 families (22 in the high risk area, 24 in the low risk area) N = 2 neighborhoods (unit not specified)	Simple content analysis	CM report (types not specified)	NBH Social process factors: child social resources, demands for social readjustment (e.g., stress) and use of helpers in response to those demands, maternal rating of family stresses and supports	In low-risk area, people were more likely to assume responsibility for child care, more likely to use the neighborhood as a resource, lower on the social readjustment scale, more likely to include professionals in list of people to call on for help, included a greater number of people listed in child’s social network, more likely to rate their neighborhood as a better place to raise children, more likely to say their children are easy to raise, rated the availability of child care higher, and more likely to engage in neighborhood exchanges.
17. Gracia & Musitu (2003) [52]		Community survey	N = 836 Families (n = 670 Non-abusive n = 166 Abusive) in Spain and Colombia	Analysis of variance	Community social support: community integration and satisfaction, community association and participation, community resources of social support (scale: the Questionnaire of Community Social Support)		In both Spain and Colombia, abusive parents had lower mean scores on community social support variables.
18. Gross-Manos et al. (2019) [53]	Environment–place duality framework	Neighborhood Factors and Child Maltreatment: A Mixed-Methods Study	N = 400 residents in 20 census tracts N = 260 child welfare workers	Multilevel mixed effects	Social disorder such as litter, loitering and social disorderly behavior. Collective efficacy including informal social control and social cohesion (developed by Sampson et al., 1997 [10])		Child welfare workers perceived higher social disorder and lower collective efficacy, compared to residents.
19. Guterman et al. (2009) [32]		2001–2003 FFCWS Wave 3 (Child age 3)	N = 3177 mothers	Structural equation modeling	Physical abuse Psychological aggression Neglect (scale: CTS-PC)	NBH social processes factors: social disorder, collective efficacy including informal social control and social cohesion (developed by Sampson et al., 1997 [10]) Mediators: parenting stress, personal control	19.1. Protective factors 19.1.1. Physical abuse: NBH social processes 19.1.2. Psychological aggression: NBH social processes 19.1.3. Neglect: none 19.2. Risk factors 19.2.1. Physical abuse: parenting stress, NBH social processes → personal control → parenting stress 19.2.2. Psychological aggression: parenting stress 19.2.3. Neglect: parenting stress, personal control, NBH social processes → personal control → parenting stress 19.3. Insignificant factors: 19.3.1. Physical abuse: personal control 19.3.2. Psychological aggression: personal control 19.3.3. Neglect: NBH social processes
20. Jespersen et al. (2022) [54]	Social capital theory	Interview data from Neighborhood Factors and Child Maltreatment: A Mixed-Methods Study	N = 113 parents in 20 census tracts	Inductive thematic approach	Interview questions How do non-kin older neighbors, as reported by neighborhood parents, contribute to neighborhood quality for families and children? In what ways might older neighbors’ contributions protect children and advance their wellbeing?		23.8% of parents reported that older neighbors provided informal social support for child wellbeing, and 27.4% reported that older neighbors promoted neighborhood safety.
21. Kim (2004) [55]	Ecological framework	1994–1994 National Longitudinal Study of Adolescent Health (Add Health) Wave 1 2000–2001 Add Health Wave 3 1990 Census	N = 2960 families in census blocks	Multilevel logistic regression	Physical abuse Neglect Any CM (aggregated both)	Individual level factors: child— irritability, health, developmental difficulty, low birth weight; parent— ethnicity, education, gender, social support, age being parents, alcohol use, drug use, self-esteem, depression, having unwanted baby, employment, physically abused as a child, neglected as a child Family level factors: number of children, single parent, violent relationship with partner, loving relationship with partner, financial support, relationship with parents NBH level factors: structural factors—ethnic heterogeneity, residential mobility, socioeconomic status, proportion single household, housing quality, violent crime rate; perceptual factors—network, happiness, safety, resources Geographical: urbanity, region	21.1. Protective factors: none 21.2. Risk factors: none 21.3. Insignificant factors 21.3.1. Physical abuse: NBH network, NBH safety, NBH resources 21.3.2. Neglect: NBH network, NBH safety, NBH resources 21.3.3. Any CM: NBH network, NBH safety, NBH resources
22. Kim & Maguire-Jack (2015) [56]	Social disorganization theory	2003–2006 FFCWS Wave 4 (child age 5)	N = 2991 mothers	Logistic regression	Physical assault Psychological aggression Neglect (scale: CTS-PC)	Community involvement: educational service available, attendance in educational service, social involvement Community perception: informal social control, social cohesion and trust (scale: Developed by Sampson et al., 1997 [10]).	22.1. Protective factors 22.1.1. Physical assualt: informal social control 22.1.2. Psychological aggression: attendance in educational service, social involvement, informal social control x social involvement 22.1.3. Neglect: none 22.2. Risk factors: nor for physical assualt, psychological aggression neglect 22.3. Insignificant factors 22.3.1. Physical assualt: educational service available, attendance in educational service, social involvement, social cohesion and trust 22.3.2. Psychological aggression: educational service available, informal social control, social cohesion and trust 22.3.3. Neglect: educational service available, attendance in educational service, social involvement, informal social control, social cohesion and trust
23. Korbin et al. (1998) [21]	Ecological perspective	Observation and interview 1991 CPS data 1990 Census	n = 94 Census tracts (predominantly African American) n = 189 Census tracts (predominantly European American) n = Informants (Numbers not specified in 4 Census tracts (two having lower CM rates, two having higher CM rates))	Ethnographic description, Content analysis	CM rates including indicated and substantiated reports of abuse and neglect	NBH Structural factors: impoverishment—family headship, poverty rate, unemployment rate, vacant housing, population loss; instability—movement, between 1985 and 1990, tenure < 10 years, recent movement in 1 year; childcare burden—child/adult ratio, male/female ratio, elderly population, contiguous to concentrated poverty Interview questions: views of NBH life— residential quality, resource availability, perceptions of physical environment, conditions of crime, danger, and drug, characteristics of neighbors	23.1. Protective factors: physical environments are quiet, clean, and peaceful; good and accessible service resources; less transient in neighborhoods; good and safe place to rear children; friendly neighbors; willing to get involved in census tracts with lower CM rates 23.2. Risk factors: lots of vacancy, trash in streets, graffiti, and vandalized buildings and cars; many temporary residents; mixed responses about resource availability; distrust and suspicion among neighbors; having problems with crime, drugs, and violence in European American NBH in census tracts with higher CM rates
24. Ma et al. (2018) [36]	Social learning theory Family coercion theory Ecological model Social disorganization theory	2001–2003 FFCWS Wave 3 (child age 3) 2003–2006 FFCWS Wave 4 (child age 5)	N = 2267 mothers	Logistic regression	Self-reported CPS involvement	Neighborhood collective efficacy: informal social control, social cohesion and trust (scale: developed by Sampson et al., 1997 [10]) Mediator: parental spanking	24.1. Protective factors: social cohesion and trust 24.2. Risk factors: none 24.3. Insignificant factors: informal social control, collective efficacy → spanking
25. Maguire-Jack & Font (2017) [57]		Community survey titled The Social Mechanisms of Child Physical Abuse and Neglect 2011–2015 American Community Survey	N = 2996 families	Multilevel logistic regression	Physical abuse including corporal punishment and severe assault (scale: CTS-PC) Neglect (scale: Multidimensional Neglect Behavior Scale)	NBH Structural factors: poverty rate, percentage of neighborhood population that moved in the past 5 years, unemployment rate, %Black, %Hispanic NBH social processes factors: reciprocated exchange, informal social control (Scale: developed by Sampson et al., 1997 [10]) Parent and family characteristics: individual poverty status, unemployment, residential instability	25.1. Protective factors 25.1.1. Physical abuse: informal social control at individual level in higher income level group 25.1.2. Neglect: informal social control at both individual and census track levels in higher income level group for supervison neglect 25.2. Risk factors: none for physical abuse and neglect 25.3. Insignificant factors 25.3.1. Physical abuse: informal social control at census tract level in higher income group 25.3.2. Neglect: reciprocated exchange at individual and census track levels in higher and lower income groups, informal social control at both individual and census track levels in higher and lower income groups for physical neglect
26. Maguire-Jack & Showalter (2016) [58]	Social disorganization theory	Community survey titled Franklin County Neighborhood Services Study	N = 896 parents	Negative binomial regression Logistic regression	Neglect: basic need neglect, neglect due to caregiver mental health or substance abuse concern (MHSA) Physical abuse: corporal punishment, severe assault (scale: CTS-PC)	Perceived NBH cohesion (scale: developed by Sampson et al., 1997 [10])	26.1. Protective factors: perceived NBH cohesion for neglect and basic need neglect 26.2. Risk factors: none 26.3. Insignificant factors: perceived NBH cohesion for physical abuse, neglect due to MHSA, corporal punishment, and severe assault
27. Maguire-Jack & Wang (2016) [59]	Developmental ecological model (Belsky, 1993) [60] Collective efficacy model (Sampson et al., 1997 [10])	Community survey	N = 1045 parents	Structural equation modeling	Neglect (scale: CTS-PC)	Perceived NBH cohesion (scale: developed by Sampson et al., 1997 [10]) Mediators: parenting stress, social support (scale: a subset of the Family Support Study)	27.1. Protective factors: social cohesion → parenting stress, social support → parenting stress, social cohesion → social support → parenting stress 27.2. Risk factors: none 27.3. Insignificant factors: perceived NBH cohesion, social support
28. Maguire-Jack et al. (2022) [7]	Social disorganization theory	2003–2006 FFCWS Wave 4 (child age 5)	N = 4898 mothers	Structural equation modeling	Physical assault Psychological aggression Neglect (scale: CTS-PC)	NBH poverty NBH social processes factors: Collective efficacy including informal social control and social cohesion and trust (scale: developed by Sampson et al., 1997 [10])	28.1. Protective factors: NBH poverty → social cohesion for physical assault and psychological aggression, NBH poverty → informal social control for neglect 28.2. Risk factors: none 28.3. Insignificant factors: NBH poverty → informal social control for physical assault and psychological aggression, NBH poverty → social cohesion for neglect
29. Manabe (2004) [61]		Neighborhood and Household Factors in the Etiology of Child Maltreatment in the National Data Archive on Child Abuse and Nelgect (Korbin & Coulton, 2002) [62] 1991–1993 CPS data 1991 Census	N = 380 households in 19 Census tracts	Regression	CM rates including reports of indicated and substantiated physical abuse, neglect, and sexual abuse	1. Poverty 2. Interaction between residents: participation in parent−child activities, use of public places, presence of relatives and friends 3. Community disorganization: interpersonal and environmental disorganization 4. Residential stability	29.1. Protective factors: use of public places 29.2. Risk factors: none 29.3. Insignificant factors: participation in parent−child activities, presence of relatives and friends
30. McLeigh et al. (2018) [63]	Social disorganization theory	Community survey 2000 Census 2000–2003 CPS data	N = 483 caregivers with children under 10 drawn from 120 Census block groups	Regression	Rates of substantiated abuse Rates of substantiated neglect	NBH poverty rate Mediator: social cohesion (Scale: Developed by Sampson et al., 1997 [10])	30.1. Protective factors 30.1.1. Abuse: social cohesion, poverty → social cohesion 30.1.2. Neglect: none 30.2. Risk factors: none for abuse and neglect 30.3. Insignificant factors 30.3.1. Abuse: none 30.3.2. Neglect: social cohesion, poverty → social cohesion
31. Merritt (2009) [64]	Social capital theory Family stress theory	Neighborhood and Household Factors in the Etiology of Child Maltreatment (Korbin & Coulton, 1999) [65] 1990 Census	N = 400 parents of children in 20 Census tracts	Hierarchical model	CAP score	NBH level factors: impoverishment (family hardship, poverty rate, unemployment rate, vacant housing, population loss, %Black), instability (movement, tenure under 10 years, recent movement), and childcare burden (child/adult ratio, male/female ratio, elderly population) Individual household factors: age, marital status, gender, employment, education, income, ethnicity, family support, and friends support (scale: Multidimensional Scale of Perceived Social Support)	31.1. Protective factors: family support, friends support
32. Molnar et al. (2013) [30]		1995 PHDCN 1995 Chicago Police crime data 1990 Census	N = 4252 Children N = 3465 Caregivers N = 343 neighborhood clusters	Hierarchical model	Parent to child physical aggression (scale: CTS)	NBH structural factors: concentrated disadvantage, immigrant concentration, residential stability, homicide rate NBH social process factors: social networks, collective efficacy including informal social control, social cohesion (scale: developed by Sampson et al., 1997 [10])	32.1. Protective factors: social network for Hispanics families 32.2. Risk factors: none 32.3. Insignificant factors: social network, collective efficacy
33. Molnar et al. (2016) [66]	Ecological framework	1995 PHDCN 1995–2005 CPS data 1995–2005 Census 1995–2005 Chicago Police Department	N = 343 NBH clusters (nested in 847 census tracts) N = 30,184 observations for 11 years	Multilevel model	Rates of indicated and substantiated neglect Rates of indicated and substantiated sexual abuse Rates of indicated and substantiated physical abuse Rates of indicated and substantiated substance-exposed infants	Collective efficacy: informal social control, social cohesion and trust Intergenerational closure Social network Physical and social disorder	33.1. Protective factors: collective efficacy, social network, and intergenerational closure for neglect, sexual abuse, and physical abuse, collective efficacy and social network for substance-exposed infants 33.2. Risk factors: physical and social disorder for neglect, sexual abuse, and physical abuse 33.3. Insignificant factors: intergenerational closure and physical and social disorder for substance-exposed infants
34. Prendergast & MacPhee (2020) [67]	Ecological framework, Risk and resilience perspectives	2001–2003 FFCWS Wave 3 (child age 3) 2003–2006 FFCWS Wave 4 (child age 5) 2007–2010 FFCWS Wave 5 (child age 9)	N = 3529 mother-child dyads	Latent growth curve modeling	Maternal aggression including physical and psychological aggression (scale: CTS-PC)	Racial/ethnic identity Baseline cumulative risk: Education Income-to-poverty ratio NBH social cohesion Parenting stress	34.1. Protective factors: social cohesion for low, modertate and high risk group at age 3
35. Seon (2021) [68]	Social disorganization theory	2001–2003 FFCWS Wave 3 (child age 3) 2003–2006 FFCWS Wave 4 2007–2010 (child age 5) 2000 Census	N = 327 foreign-born mothers in 325 census tracts	Structural equation modeling by complex model	Physical assault Psychological aggression Neglect (scale: CTS-PC)	1. NBH structural factors: poverty, vacant housing units, foreign-born residents, household on public assistance, unemployment, families headed my females 2. NBH social processes factors: collective efficacy including informal social control and social cohesion and trust (scale: developed by Sampson et al., 1997 [10]), community participation	35.1. Protective factors: NBH social processes for physical assualt and psychological aggression, NBH structural factors → NBH social processes for physical assault 35.2. Risk factors: NBH structural factors for physical assualt, psychological aggression, and neglect 35.3. Insignificant factors: NBH social processes for neglect, NBH structural factors → NBH social processes for psychological aggression and neglect
36. Spilsbury et al. (2022) [69]		Neighborhood Factors and Child Maltreatment: A Mixed-Methods Study	N = 400 caregivers in 20 census tracts		Responses to child in need (i.e., series of five scenarios involving a situation of a child potentially in need, such as being abused or neglected, or a child misbehaving)	Length of residence Social network size Collective efficacy including informal social control and social cohesion (scale: developed by Sampson et al., 1997 [10]) Retaliation (degree to which caregivers perceived a risk of verbal or physical retaliation for intervening in situations involving children) Victimization (degree to which residents worry about becoming victims of crime and violence)	36.1. Protective factors: collective efficacy, collective efficacy × victimization
37. Spilsbury et al. (2018) [70]		Community survey	N = 60 adult caregivers in Cleveland N = 60 adult caregivers in Tel Aviv	Logistic regression	Agreement that neighbors can do something about CM		37.1. Protective factors: none 37.2. Risk factors: none 37.3. Insignificant factors: endorsing the statement about neighbors can do something about CM between caregivers in Cleveland and Tel Aviv
38. Tajima & Harachi (2010) [71]	Intersectionality theory	2002 The Cross-Cultural Families project	N = 327 mothers	Logistic regression	Breaking the intergenerational cycle of physical discipline (=of those parents who had been hit by either parent, those who did not report using any physical discipline with their own child)	Caregiver (mother) factors: education, acculturation to United States, depression, age, personal support system Household factors: financial insecurity, neighborhood support, partner in household, Cambodian/Vietnamese group Child factors: child behavior problems, age, gender	38.1. Protective factors: none 38.2. Risk factors: none 38.3. Insignificant factors: NBH support
39. Vinson et al. (1996) [72]	Ecological framework	Interview	N = 97 n = 51 Adults in the southern areas (high CM risk), n = 46 in the northern comparison areas (low CM risk) in 2 Collector’s Districts (Australian census units)	Analysis of variance	Description of NBH such as sources of help and the presence of mutual support, locality as a place to raise children Membership of carer’s support network Indicate who they would talk to about five kinds of problems (personal, money, child rearing, household and work/education)	Group with high-risk of CM Group with low-risk of CM	39.1. Protective factors: larger numbers of neighbors and acquaintance, higher level of across-network interaction (home-acquaintances, home-neighbors, close family-acquaintances, close friends and acquaintances) in low-risk area 39.2. Risk factors: none 39.3. Insignificant factors: locality as a place to raise children
40. Wang et al. (2019) [73]		Community survey	N = 1337 parents	Logistic regression	Physical violence Minor physical violence Severe physical violence (scale: CTS-PC)	Perception of NBH collective efficacy including informal social control and social cohesion and trust (scale: developed by Sampson et al., 1997 [10])	40.1. Protective factors: social cohesion for physical violence and minor physical violence 40.2. Risk factors: none 40.3. Insignificant factors: informal social control for physical violence, minor physical violence, and severe physical violence, social cohesion for severe physical violence
41. Wolf (2015) [74]		Community survey	N = 3023	Multilevel models	Physical abuse (scale: CTS-PC)	Social support including tangible, emotional support, companionship (scale: Interpersonal Support Evaluation List) Collective efficacy including informal social control and social cohesion (scale: developed by Sampson et al., 1997 [10])	41.1. Protective factors: emotional support 42.2. Risk factors: none 41.3. Insignificant factors: tangible support, companionship, informal social control, social cohesion

^a^ Analytical approach reported in table is a method to analyze social processes factors, ^b^. → indicates a mediational path, ^c^. × indicates an interaction between two variables.

## Data Availability

Not applicable.
